# Human Synaptic Plasticity Gene Expression Profile and Dendritic Spine Density Changes in HIV-Infected Human CNS Cells: Role in HIV-Associated Neurocognitive Disorders (HAND)

**DOI:** 10.1371/journal.pone.0061399

**Published:** 2013-04-19

**Authors:** Venkata Subba Rao Atluri, Sudheesh P. Kanthikeel, Pichili V. B. Reddy, Adriana Yndart, Madhavan P. N. Nair

**Affiliations:** Department of Immunology, Institute of NeuroImmune Pharmacology, Herbert Wertheim College of Medicine, Florida International University, Miami, Florida, United States of America; University of Nebraska Medical Center, United States of America

## Abstract

HIV-associated neurocognitive disorders (HAND) is characterized by development of cognitive, behavioral and motor abnormalities, and occur in approximately 50% of HIV infected individuals. Our current understanding of HAND emanates mainly from HIV-1 subtype B (clade B), which is prevalent in USA and Western countries. However very little information is available on neuropathogenesis of HIV-1 subtype C (clade C) that exists in Sub-Saharan Africa and Asia. Therefore, studies to identify specific neuropathogenic mechanisms associated with HAND are worth pursuing to dissect the mechanisms underlying this modulation and to prevent HAND particularly in clade B infection. In this study, we have investigated 84 key human synaptic plasticity genes differential expression profile in clade B and clade C infected primary human astrocytes by using RT^2^ Profile PCR Array human Synaptic Plasticity kit. Among these, 31 and 21 synaptic genes were significantly (≥3 fold) down-regulated and 5 genes were significantly (≥3 fold) up-regulated in clade B and clade C infected cells, respectively compared to the uninfected control astrocytes. In flow-cytometry analysis, down-regulation of postsynaptic density and dendrite spine morphology regulatory proteins (ARC, NMDAR1 and GRM1) was confirmed in both clade B and C infected primary human astrocytes and SK-N-MC neuroblastoma cells. Further, spine density and dendrite morphology changes by confocal microscopic analysis indicates significantly decreased spine density, loss of spines and decreased dendrite diameter, total dendrite and spine area in clade B infected SK-N-MC neuroblastoma cells compared to uninfected and clade C infected cells. We have also observed that, in clade B infected astrocytes, induction of apoptosis was significantly higher than in the clade C infected astrocytes. In conclusion, this study suggests that down-regulation of synaptic plasticity genes, decreased dendritic spine density and induction of apoptosis in astrocytes may contribute to the severe neuropathogenesis in clade B infection.

## Introduction

HIV is a neurotropic virus that directly invades the brain shortly after infection. HIV replicates in brain macrophages and microglia causing inflammatory and neurotoxic host responses. HIV can also cause severe neurological disorders, collectively known as HIV-associated neurocognitive disorders (HAND). HAND is characterized by development of cognitive, behavioral and motor abnormalities. HIV-1 displays wide genetic variation in global distribution. It is classified into three groups (M, O and N) and genetically into nine different subtypes (A–K). Of these, clades B and C represent the majority (>86%) of circulating HIV-1 variants [1]. Clade B is predominant in North America, Western Europe, and Australia, whereas clade C is common in Africa, Latin America, and Asia. Prior to the widespread use of highly active antiretroviral therapy (HAART), 20–30% of individuals with advanced HIV-1 clade B infection displayed symptoms of the most severe HAND disorder, HIV-associated dementia (HAD) [2], [3]. On the contrary, Satischandra et al (2000) [4] and few other studies [5] have reported unusually very low incidence-about 1–2% of HAD in HIV-1 clade C infected patients from India. Since the widespread use of HAART, the incidence of HAD has dramatically decreased; however, as many as 40–50% of HIV-positive patients continue to suffer from HAND [6], [7], [8], [9], [10]. Since autopsies are seldom performed in developing countries where HIV-1 infection is mainly with clade C, our current understanding of the pathophysiology and neuropathology of HIV-1 infection emanated mainly from clade B. Therefore, very little information is available on neuropathogenesis of clade C. It has been reported that replication efficiency of clade C in in-vitro monocyte-derived macrophages is significantly less than clade B isolates [11]. In the same study, authors have also reported less neurotoxicity in clade C infected neuronal cells than clade B infected cells.

Microglia/macrophages are the most commonly infected cells in the brain and serve as lifelong hosts for HIV [2], [12], [13]. A second cellular target for HIV in the brain is astrocytes [14], [15], [16]. Astrocytes are the most abundant cell type in the brain [17] and perform many essential functions, such as maintenance of a homeostatic environment, bidirectional communication with neurons [18] and immune functions within the nervous system. The extensive synaptic interaction not only ensures that astrocytes are able to fulfill their metabolic support roles but also positions astrocytes to directly influence the structure and function of the synapse. Indeed there is an increasing evidence that astrocytes play an active role in controlling the number and strength of a neuron's synapses and thus may contribute to mechanisms underlying synaptic plasticity [19]. Therefore, use of astrocytes in the HIV infection studies may lead to better understanding of the neuropathogenesis of HIV.

The brain recalls immediate events via short-term memories; however, it must consolidate these events into long-term memory for later recall. Memory consolidation requires synaptic plasticity characterized by physical changes to, and gene expression changes in, neuronal synapses. Synaptic plasticity studies have discovered immediate-early genes (IEGs) that alter expression immediately after neuronal events. IEGs mediate long-term potentiation (LTP), a process that enhances synaptic connections and consolidates memories. However, as not all events become long-term memories, the opposite synaptic remodeling response, long-term depression (LTD), also plays a central role in synaptic plasticity. Gene expression changes associated with LTD yield physical changes in the neuronal synapse that recycle receptors and either enhance or inhibit synaptic connections. The synaptic plasticity genes are categorized into different groups (Table 1). We hypothesize that infection of clade B and C differentially express the synaptic plasticity genes, alters the dendrite morphology and also induce apoptosis in brain cells.

**Table 1:** Functional Grouping of Human Synaptic Plasticity genes.

**1. Immediate-Early Response Genes (IEGs)**	ARC, BDNF, CEBPB, CEBPD, CREB1, CREM, EGR1, EGR2, EGR3, EGR4, FOS, HOMER1, JUN, JUNB, KLF10, MMP9 (Gelatinase B), NFKB1, NFKBIB (TRIP9), NGF, NPTX2, NR4A1, NTF3, PCDH8, PIM1, PLAT (tPA), RELA, RGS2, RHEB, SRF, TNF
**2. Late Response Genes**	INHBA, SYNPO.
**3. Long Term Potentiation (LTP)**	ADCY1, ADCY8, BDNF, CAMK2A, CAMK2G, CDH2 (N-cadherin), CNR1, GABRA5, GNAI1, GRIA1, GRIA2, NMDAR1 (GRIN1/NR1), GRIN2A, GRIN2B, GRIN2C, GRIN2D, MAPK1, MMP9 (Gelatinase B), NTF4, NTRK2, PLCG1, PPP1CA, PPP1CC, PPP3CA, PRKCA, PRKCG, RAB3A, YWHAQ (14-3-3).
**4. Long Term Depression (LTD)**	GNAI1, GRIA1, GRIA2, GRIA3, GRIA4, GRIP1, GRM1, GRM2, IGF1, MAPK1, NOS1, NGFR, PICK1, PLAT (tPA), PPP1CA, PPP1CC, PPP1R14A (CPI-17), PPP2CA, PPP3CA, PRKCA, PRKG1
**5. Cell Adhesion**	ADAM10, CDH2 (N-cadherin), GRIN2A, GRIN2B, NCAM1, PCDH8, PPP2CA, RELN, TNF.
**6. Extracellular Matrix & Proteolytic Processing**	ADAM10, MMP9 (Gelatinase B), PLAT (tPA), RELN, TIMP1
**7. CREB Cofactors**	AKT1, CAMK2G, NMDAR1, GRIN2A, GRIN2B, GRIN2C, GRIN2D, MAPK1 (ERK2), PPP1CA, PPP1CC.
**8. Neuronal Receptors**	EPHB2, GABRA5, GRIA1, GRIA2, GRIA3, GRIA4, NMDAR1, GRIN2A, GRIN2B, GRIN2C, GRIN2D, GRM1, GRM2, GRM3, GRM4, GRM5, GRM7, GRM8, NTRK2.
**9. Postsynaptic Density**	ADAM10, ARC, DLG4 (PSD95), GRIA1, GRIA3, GRIA4, NMDAR1, GRIN2A, GRIN2B, GRIN2C, GRM1, GRM3, HOMER1, PICK1, SYNPO.
**10. Others**	KIF17, SIRT1.

In this study, we have analyzed the expression of 84 human synaptic plasticity genes central to synaptic alterations during learning and memory in HIV-1 clade B and C infected primary human astrocytes using human Synaptic Plasticity RT^2^ Profile PCR Array. Till now, there are no reports of analysis of synaptic plasticity gene expression profile in response to clade B and C infection. This array includes IEGs and other genes important for LTP and LTD, as well as key neuronal receptor genes and genes important for synapse remodeling. From the list of down-regulated synaptic density genes, we have further analyzed the expression of respective proteins which plays an important role in the maintenance of dendritic architecture and spine density i.e activity regulated cytoskeleton protein (ARC), Glutamate receptor 1 (GRIN1)/N-methyl-D-aspartate receptors subunit 1 (NMDAR1) and Glutamate receptor metabotropic 1 (GRM1) in both clade B and clade C infected astrocytes and SK-N-MC neuroblastoma cells. Dendritic spines are the postsynaptic specializations and believed to regulate the strength of synaptic transmission and play critical role in neuronal plasticity. We have also observed the significant spine density and dendrite morphology changes in HIV-1 clade B and C infected SK-N-MC cells using confocal microscopy. In HIV infected patients, altered dendritic spine density may also attribute for the increased incidence of neurocognitive disorders (HAND). Induction of apoptosis in HIV-1 infected neuronal cells has been reported earlier [20]. In this study, we have found that the induction of apoptosis was significantly higher in clade B infected astrocytes than in clade C infected cells.

## Materials and Methods

### Cell culture and reagents

Primary human astrocytes were purchased from ScienCell Research laboratories (Carlsbad, CA; Cat. # 1800-5) and grown in astrocyte medium purchased from ScienCell laboratories (Cat. # 1801) containing 2% of fetal bovine serum (ScienCell Cat. # 0010), astrocyte growth supplement (ScienCell Cat. # 1852) and penicillin/streptomycin (ScienCell Cat. # 0503). Human neuroblastoma SK-N-MC cells were obtained from ATCC (ATCC Cat # HTB-10). HIV-1_Ba-L_ (clade B) (NIH AIDS Reagent Program Cat. # 510) and HIV-1-98CN006 (clade C) (NIH AIDS Reagent Program, Cat. # 4164) were obtained through AIDS Research and Reference Reagent Program, Division of AIDS, NIAID, NIH.

### HIV-1 infection of primary human astrocytes and SK-N-MC human neuroblastoma cells

Primary human astrocytes and SK-N-MC human neuroblastoma cells were infected with HIV-1 using the previously described protocol [21], [22] with slight modifications. Briefly, astrocytes (1×10^6^ cells) and SK-N-MC (1×10^6^ cells) cells were cultured overnight in T-75 flasks using astrocyte medium and minimum essential medium, respectively. The cells were activated by treating with polybrene (10 µg/ml) for 6–7 hrs before the infection. The cells were infected with TCID50 of HIV-1 clade B and C virus (dose that produces approximately same levels of P24 antigen in culture) for 7–10 days under same experimental conditions. On every second day, half of the medium was replaced with the fresh medium and the supernatant obtained from the used medium was used for the p24 antigen estimation using ELISA kit (ZeptoMetrix Corp. Cat # 0801200). Controls cells (without clade B or clade C) were included in the set-up of all experiments.

### mRNA isolation and first strand cDNA synthesis

After 7 days of clade B/clade C infection, primary human astrocytes were harvested and the pellet was used for the mRNA isolation using illustra triplePrep Kit (GE Healthcare Life Sciences, UK; Cat # 28-9425-44) and on-column DNase treatment step was also performed in the procedure. Purity of the RNA was measured by microspot RNA reader (Synergy HT Multi-Mode Microplate Reader from BioTek, US) and RNAs with an OD260 nm/OD280 nm absorbance ratio of at least 2.0 were used for PCR array. This mRNA was also used for the long terminal repeat (LTR)-gene expression using RT-qPCR to measure the HIV infectivity. One microgram of RNA (Control, clade B and clade C infected) was used for the first strand cDNA synthesis using SABiosciences's RT^2^ First Strand Kit (Cat # 330401) as per supplier's protocol. Genomic DNA elimination step was performed before going for reverse transcription.

### Human Synaptic Plasticity RT^2^ Profile PCR Array

Synaptic plasticity gene profiling was done using 96 well format RT^2^ Profile PCR Array human Synaptic Plasticity kit (SABiosciences, Cat # PAHS-126A-2) using Stratagene Mx3000p qRT-PCR instrument. The human Synaptic Plasticity RT^2^ Profiler PCR Array interrogates 84 genes related to the human synaptic plasticity. This kit was chosen because it includes diverse genes important in the human synaptic plasticity, including Immediate-Early Response (n = 30), Late Response (n = 2), Long Term Potentiation (n = 28), Long Term Depression (n = 21), Cell Adhesion (n = 9), Extracellular Matrix & Proteolytic Processing (n = 5), CREB Cofactors (n = 10), Neuronal Receptors (n = 19), Postsynaptic Density (n = 15), as well as other genes involved in the synaptic plasticity (n = 2). Few genes have role in multiple functions listed above. The array was performed in three independent set of experiments. Relative abundance of each mRNA species was assessed using RT^2^ SYBR Green/ROX PCR Master mix (SABiosciences, Cat # 330520) and aliquoted in equal volumes (25 µl) to each well of the real-time PCR arrays. The real-time PCR cycling program (as indicated by the manufacturer) was run on a Stratagene Mx3000p qRT-PCR thermal cycler. The threshold cycle (Ct) of each gene was determined by using the Stratagene MaxPro software. The threshold and baseline were set manually according to the manufacturer's instructions. Ct data were uploaded into the data analysis template on the manufacturer's website (http://pcrdataanalysis.sabiosciences.com/pcr/arrayanalysis.php). The relative expression of each gene in clade B or clade C infected primary human astrocytes was calculated using ΔΔCT method with five housekeeping genes and compared with the expression in control cells. Controls are also included on each array for genomic DNA contamination, RNA quality, and general PCR performance.

### Flow-cytometry

In another set of experiment, astrocytes and SK-N-MC neuroblastoma cells were infected with HIV-1 clade B and C. Cells were probed with flow cytometry antibodies for dendritic spine density and dendritic architecture regulated proteins i.e ARC (Bioss antibodies, Cat # bs-0385R-PE-Cy5), NMDAR1 (GRIN1/NR1) (Santa Cruz Biotechnology, Cat # sc-1467-PE) and GRM1 (Bioss antibodies, Cat # bs-1803R-AF647). In brief, cells were washed with FACS buffer and probed with the respective antibodies as indicated in the user manual and incubated at room temperature (RT) for 20 min in the dark. Probed cells were washed with the FACS buffer and fixed with 2% paraformaldehyde and analyzed by FACScalibur within 1 hr.

### Measurement of spine density and dendrite morphology

#### DiI staining

Established protocols to stain the neuronal cells and measurement of the spine density were used with few modifications [23], [24], [25]. In brief, SK-N-MC neuroblastoma cells were grown in Eagle's minimal essential medium containing 10% fetal bovine serum, 5 mM sodium pyruvate, 100 units/ml penicillin, 100 mg/ml streptomycin and retinoic acid at 37°C with 5% CO_2_. SK-N-MC cells were grown onto 22 mm×50 mm glass coverslips placed in a petri-dish. Cells were treated with polybrene for 8 hrs followed by addition of clade B/C virus. After 7 days of infection, cells were fixed with 4% Formaldehyde in PBS for 30 min at RT. The fluorescent membrane tracer 1, 1′-Dioctadecyl-3, 3,3′,3′-tetramethylindocarbocyanine perchlorate (DiI) at 7.5 µg/ml (in PBS) concentration was directly added onto the fixed cultures and allowed to incubate for 90 min at RT. These stained coverslips were placed overnight at 4°C in petri dishes containing PBS before proceeding for confocal microscopy.

#### Confocal Microscopy

Confocal images were obtained using TCS SP2 Confocal Laser Scanning Microscope (Leica Microsystems, Germany) at 488 nm (100%) illusion of an argon-ion laser using 60X oil immersion objectives with high numeric aperture and 2.5X confocal electronic zoom settings to visualize individual cells and dendrites. Twenty Optical serial sections of 0.14 µm/section (∼2.8 µm total) through the cells were captured and reconstructed to yield complete “two dimensional” images of individual cells in focus.

### Detection of Apoptosis

Seven days after infection with clade B and C, primary human astrocytes were washed twice with cold PBS and then re-suspended in 1X binding buffer at a concentration of 1×10^6^ cells/ml. From this, 100 µl was added to 5 ml FACS tubes, followed by incubation with 5 µl each of Annexin V and 7-AAD for 15 minutes at RT in the dark. After incubation, 400 µl of 1X binding buffer was added to each tube, mixed gently and analyzed by FACScalibur within 1 hr. The untreated population was used for defining the basal level of apoptotic and dead cells. The percentage of cells that have been induced to undergo apoptosis was then determined by subtracting the percentage of apoptotic cells in the untreated population from percentage of apoptotic cells in the treated population. Cells treated with camptothecin for 5 hrs at 37°C was used as positive control.

### Data Analysis

In the expression studies, a gene was considered differentially regulated if the difference was ≥3 fold in comparison with the control. Experiments were performed at least three times and the values obtained were averaged. All the results were expressed as mean ± s.e.m. Statistical analysis of two groups was performed by Student's t test, while more than two groups were analyzed using one way ANOVA followed by Bonferroni's multiple comparison test. Differences were considered significant at p≤0.05. Data analysis was performed with the Statistical Program, GraphPad Prism software (La Jolla, CA).

**Figure 1:**
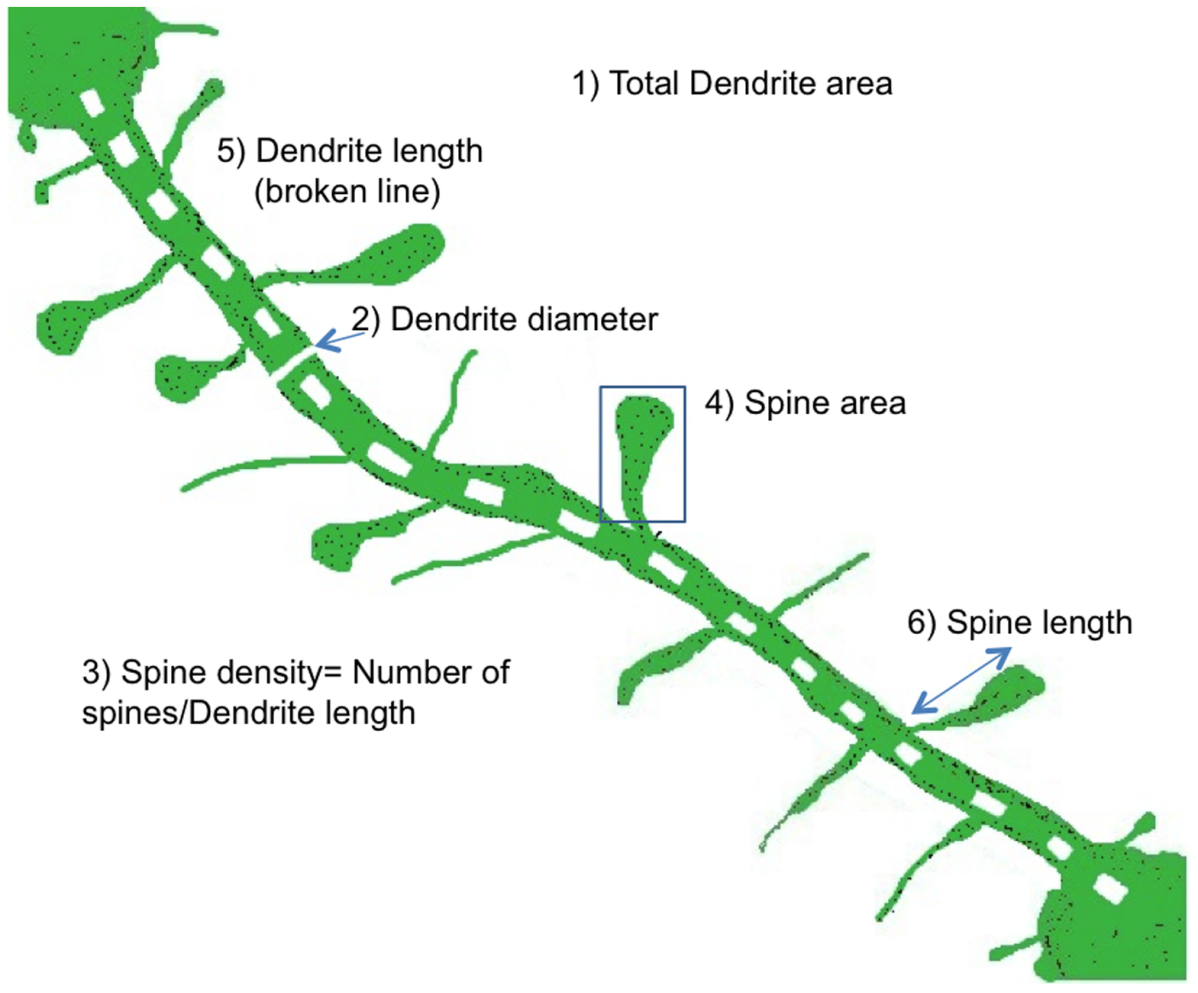
Neuronal dendrite and spine measurement by Image J analysis. A typical dendrite segment from a pyramidal neuron is shown, and the six quantification parameters labeled as follows. 1) Total dendrite area is measured by drawing a box around the whole image; 2) dendrite diameter is obtained by drawing a line across the dendrite thickness at a place of average width; 3) spine density is the total number of spines divided by the dendrite length; 4) spine area is measured by drawing a box around the whole spine; 5) Dendrite length uses the broken line tool to measure the length and 6) Spine length. (Smith et al., 2009. Reversal of long-term dendritic spine alterations in Alzheimer disease models. Proc Natl Acad Sci U S A 106: 16877-16882. Copyright (2009) National Academy of Sciences, U.S.A.)

ImageJ software program was used to quantify DiI-labeled cells (Figure 1). Dendritic segments were chosen randomly from the apical and basal regions and at least one soma's length away from the cell soma. Six parameters were measured, including total dendrite area, dendrite diameter, spine density (number of spines divided by the dendrite length), spine area and spine length [25].

## Results

### Human Synaptic plasticity genes expression in primary human astrocytes after infection with clade B and clade C

**Figure 2:**
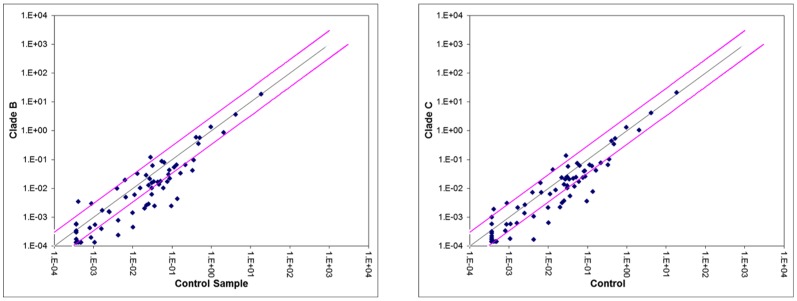
a. Scatter plot analysis of the changes in synaptic plasticity gene expression in clade B infected astrocytes. Pair wise comparison of control primary human astrocytes (No HIV infection) and clade B infected astrocytes by scatter plot analysis. Spots associated with individual human synaptic plasticity gene were collected and converted into log10 scale. The central line indicates unchanged gene expression. The synaptic plasticity genes with expression levels higher or lower in clade B infected astrocytes than the control cells are expected to produce dots that deviate from the centerline. **b. Scatter plot analysis of the changes in synaptic plasticity gene expression in clade C infected astrocytes.** Pair wise comparison of control primary human astrocytes (No HIV infection) and clade C infected astrocytes by scatter plot analysis. Spots associated with individual human synaptic plasticity gene were collected and converted into log10 scale. The central line indicates unchanged gene expression. The dots are allocated to positions that are above or below than the +3 fold or −3 fold line when the differences are greater than three folds.

Total 84 key human synaptic plasticity genes were analyzed in the RT^2^ Profile human Synaptic Plasticity PCR Array and fold change in the gene expression profile in clade B and clade C infected astrocytes was analyzed. The results were plotted in scatterplot analysis graph (Figure 2a and 2b). Out of 84 genes investigated, total 21 genes were significantly (≥3 fold) down-regulated (Table 2) in clade C infected cells compared to control cells and 5 synaptic plasticity genes (EGR2, EGR4, HOMER1, INHBA, SYNPO) were significantly up-regulated (Table 3) in both clade B and C infected cells. In clade B infected cells, along with the genes down-regulated in clade C infected cells, additionally 10 other synaptic plasticity genes (total 31 genes) were also down-regulated than control uninfected cells (Table 2). All these genes were functionally categorized into 10 groups. The fold down regulations for clade B affected genes were higher (range 3–33 fold) compared to clade C affected genes (range 3–28 fold) with respect to control.

**Table 2:** Human synaptic plasticity genes down-regulated in HIV-1 clade B & C infected primary human astrocytes (fold down regulation): Out of 84 genes analyzed, only the genes significantly (≥3 fold) down-regulate were shown in this table.

Synaptic plasticity genes	Clade B fold change	Clade C fold change	p-value
ADAM10^4^^,^ 5 ^,^ 8	−3.5	−2.9	<0.005
AKT1^6^	−15.31	−6.8	<0.001
ARC^1^^,^ 8	−3.2	−2.6	<0.024
CAMK2A^2^	−3.7	−3.4	<0.001
CDH2^2^^,^ 4	−3.8	−3.5	<0.018
CEBPB^1^	−4.3	−2.7	<0.001
CEBPD^1^	−8.3	−6.2	<0.001
CNR1^2^	−9.4	−7.3	<0.001
CREB1^1^	−3.5	−2.1	<0.001
DLG4^8^	−9	−7.4	<0.001
EGR1^1^	−3.1	−2.1	<0.002
FOS^1^	−8.3	−5.1	<0.001
GABRA5^2^^,^ 7	−3.5	−4	<0.002
GRIA1^2^^,^ 3 ^,^ 7 ^,^ 8	−41	−28	<0.001
NMDAR1 (GRIN1/NR1)^2^^,^ 6 ^,^ 7 ^,^ 8	−3.2	−2.6	<0.024
GRIN2B^2^^,^ 4 ^,^ 6 ^,^ 7 ^,^ 8	−4.7	−1.6	<0.001
GRM1^3^^,^ 7 ^,^ 8	−3.1	−2.7	<0.024
GRM8^7^	−3.6	−3.4	<0.020
JUN1^1^	−5.1	−3.1	<0.001
JUNB^1^	−7.3	−4.8	<0.002
MAPK1^2^^,^ 3 ^,^ 6	−5.1	−4.1	<0.005
NFKBIB^1^	−3.1	−2.9	<0.001
NGFR^3^	−23.2	−16.6	<0.001
NPTX2^1^	−18.7	−27	<0.001
PICK1^3^^,^ 8	−5.7	−4.2	<0.004
PLCG1^2^	−10.2	−9.3	<0.001
PPP1CA^2^^,^ 3 ^,^ 6	−32.6	−18.3	<0.001
PRKCA^2^^,^ 3	−4.7	−3.4	<0.001
RELA^1^	−4.1	−3.5	<0.001
SIRT1^9^	−3.1	−2.5	<0.001
SRF^1^	−6	−3.7	<0.001

1 Immediate-Early Response Genes (IEGs);

2 Long Term Potentiation (LTP);

3 Long Term Depression (LTD);

4 Cell Adhesion;

5 Extracellular Matrix & Proteolytic Processing;

6 CREB Cofactors;

7 Neuronal Receptors;

8 Postsynaptic Density;

9 Others.

**Table 3:** Human synaptic plasticity genes up-regulated in HIV-1 clade B & C infected primary human astrocytes (fold up regulation).

Gene	clade B	clade C
EGR2^1^	3.1	3.2
EGR4^1^	8.3	4.4
HOMER1^1^^,^ 3	3	3
INHBA^2^	4	4.5
SYNPO^2^^,^ 3	3	3.2

1 Immediate-Early Response Genes (IEGs);

2 Late Response Genes;

3 Postsynaptic Density.

### Decreased postsynaptic density regulated proteins (ARC, NMDAR1 and GRM1) in CNS cells

**Figure 3:**
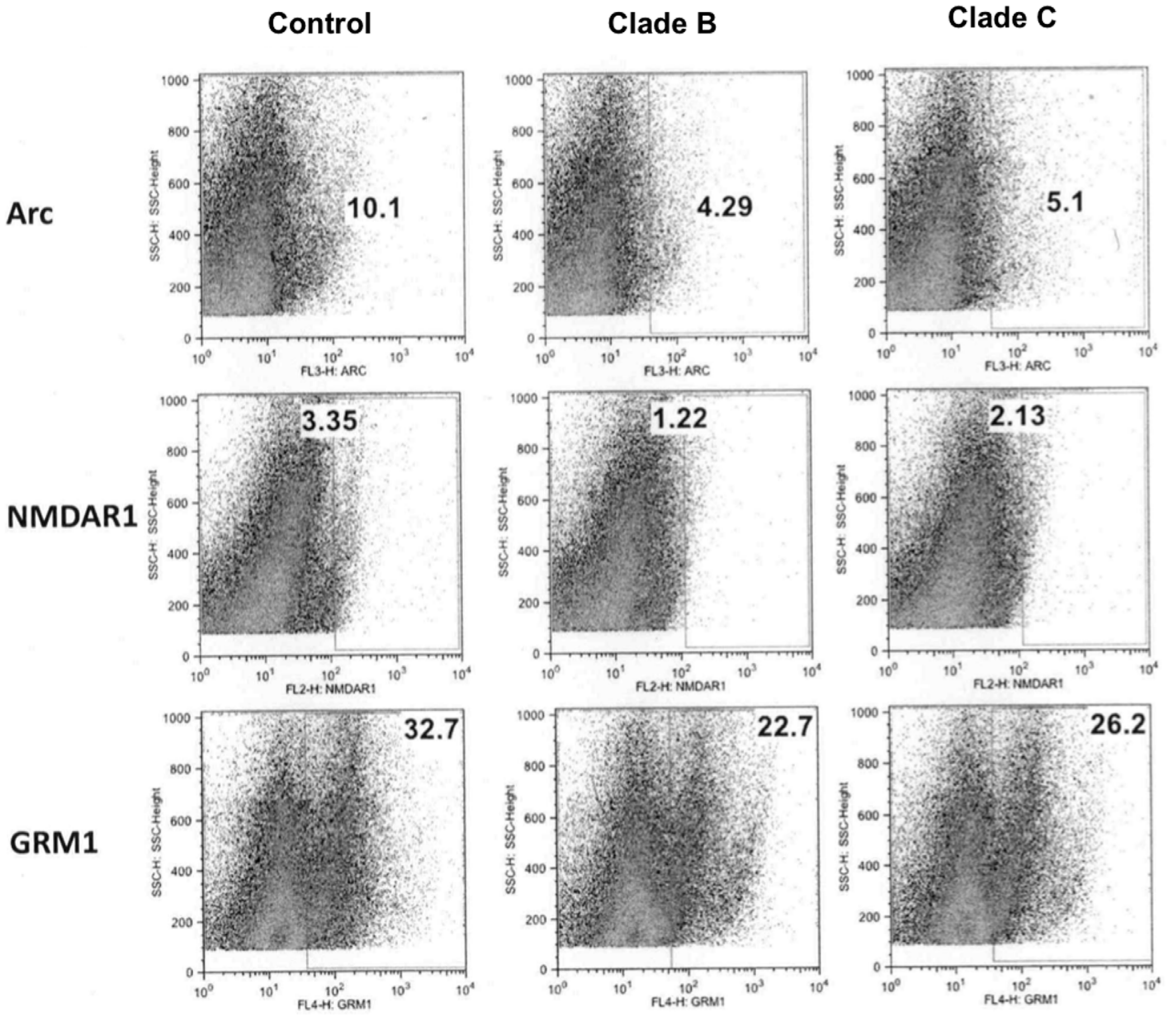
Flow-cytometry analysis of postsynaptic density and dendrite morphology regulatory proteins. Levels of ARC, NMDAR1 and GRM1 proteins were measured in clade B and C infected astrocytes by flow-cytometry. The expression levels of these three proteins were significantly less in clade B infected astrocytes than clade C (p<0.02) infected and uninfected control cells (p<0.001).

In flow cytometry analysis, expression levels of synaptic density and dendritic architecture regulating proteins ARC (p<0.001 and 0.02), NMDAR1 (p<0.007 and 0.005) and GRM1 (p<0.006 and 0.04) were significantly decreased in HIV-1 clade B infected primary human astrocytes than the control and clade C infected cells, respectively. In clade C infected cells as well, these three proteins (ARC, NMDAR1 and GRM1) were significantly down-regulated than control astrocytes (p<0.02, 0.006, 0.01, respectively). Representative figures showing differential protein expression were presented in Figure 3. Similarly, we have also observed significantly decreased expression of ARC, NMDAR1 and GRM1 proteins in clade B and C infected SK-N-MC neuroblastoma cells than the control cells (data not shown).

### Altered dendritic architecture in clade B and clade C infected SK-N-MC cells

**Figure 4:**
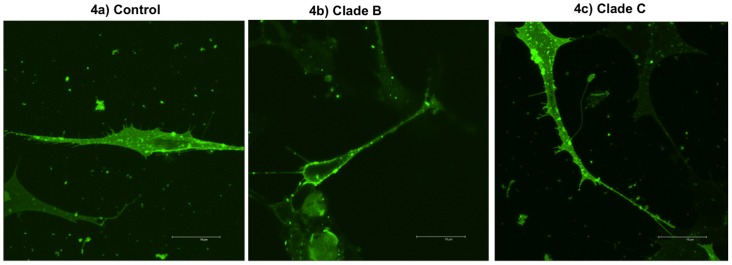
Confocal Images of DiI stained SK-N-MC cells. (4a) Control SK-N-MC cells: High number of long spines on the dendritic length, high dendrite diameter, total dendrite area and spine area. (4b) Clade B infected cells: Loss of spines on the dendrite length, decreased dendrite diameter, dendrite and spine area was observed than the control and clade C infected SK-N-MC cells. (4c) Clade C infected cells: Loss of number of spines, decreased dendrite diameter, dendrite area and spine area was observed than control cells.

**Figure 5:**
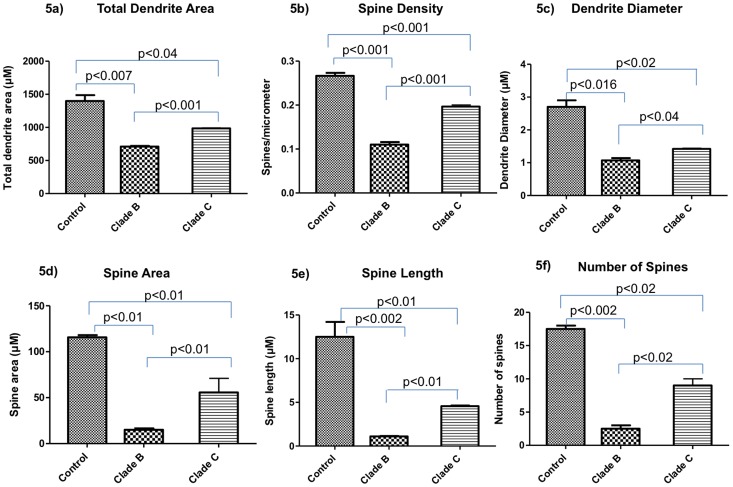
Dendrite morphology changes in HIV-1 clade B and clade C infected SK-N-MC neuroblastoma cells. SK-N-MC neuroblastoma cells were grown onto the glass coverslips and infected with the HIV clade B and C for 7 days. Cover slips were stained with the DiI stain and observed under confocal microscopy. Randomly selected pictures in each group of the cells were captured in confocal microscope. Image J software was used to analyze the total dendrite area (5a), spine density (5b), dendrite diameter (5c), spine area (5d), spine length (5e) and number of spines (5f).

SK-N-MC dendrite morphology of control (Fig. 4a), HIV-1 clade B (Fig. 4b) and clade C (Fig. 4c) infected cells were captured using confocal microscopy and morphological changes were analyzed using the established protocol [25]. Infection of SK-N-MC cells with clade B and C resulted in significant decrease in total dendrite area (p<0.007, p<0.04) (Figure 5a), spine density (p<0.001, p<0.001) (Fig. 5b), dendrite diameter (p<0.016, p<0.02) (Fig. 5c), spine area (p<0.01, p<0.01) (Fig. 5d), spine length (p<0.002, p<0.01) (Fig. 5e) and number of spines (p<0.002, p<0.02) (Fig. 5f) compared with uninfected control cells, respectively. In clade B infected cells, except dendrite length (p<0.16), significant decrease in dendrite area (p<0.001), spine density (p<0.001), dendrite diameter (p<0.04), number of spines (p<0.02), spine length (p<0.01) and spine area (p<0.01) was observed than with clade C infected cells.

### Induction of apoptosis in HIV-1 clade B and clade C infected astrocytes

**Figure 6:**
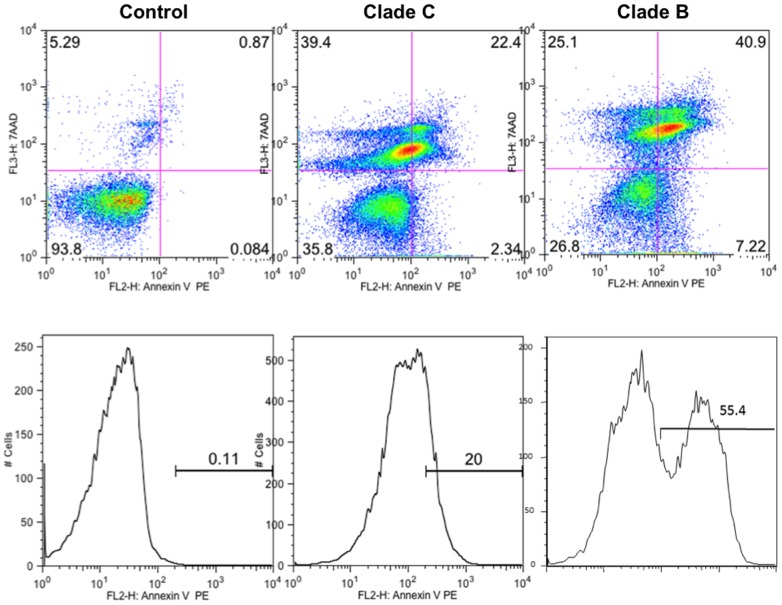
Apoptosis induction in HIV infected primary human astrocytes. Apoptosis induction was studied using double cell labeling with Annexin V-PE and 7-AAD. Seven days after infection, cells were collected and re-suspended in binding buffer containing Annexin V-PE and 7-AAD, and then processed for flow cytometry analysis. Representative flow figure for one of three experiments is shown here. In each box, cells in lower left corner (negative for 7-AAD and Annexin V-PE) are viable, cells in the upper right corner (positive for 7-AAD and Annexin V-PE) are necrotic or late apoptotic cells, while cells in the lower right corner (Annexin V-PE positive but negative to 7-AAD) are early apoptotic cells.

As shown in Figure 6, the percentages of apoptotic cells were significantly higher in both clade B (41%, p<0.001) and clade C infected cells (22.4%, p<0.003) compared to uninfected cells. Compared to the clade B infected cells, apoptosis induction was significantly lower (p<0.005) in clade C infected cells. Taken together, these results suggested that the clade B infection being the most neurotoxic to primary human astrocytes than clade C.

## Discussion

In this study, in clade B infected astrocytes, we have observed down-regulation of 31 key synaptic plasticity genes and dysregulation of many other synaptic plasticity genes than the control and clade C infected cells. In clade C infected astrocytes, total 21 synaptic plasticity genes (also down-regulated in clade B infected cells) were down-regulated compared to the control cells. It indicates that clade B is more neuropathogenic than clade C by down-regulating the more number of synaptic plasticity genes and in higher folds. Interestingly five genes (EGR2, EGR4, HOMER1, INHBA, SYNPO) were significantly up regulated in both clade B and C infected astrocytes compared to uninfected astrocytes and the significances of these genes are yet to be elucidated. Gene profiling studies have shown that HIV-Tat down-regulates genes involved in the Ras-Raf-MEK1 signaling [26], a pathway involved in modulating synaptic plasticity [27]. In one of the gene dysregulation studies in HIV encephalitis patients, down-regulation of synaptic plasticity genes has been reported [28]. Recently from our lab, increased levels of HDAC2 in HIV-1 Tat treated SK-N-MC cells was reported [29]. Chromatin remodeling due to the increased levels of HDAC2 may explain the differential expression of synaptic plasticity genes in HIV infected cells. Each synaptic gene we have studied has one or more synaptic functions among the 10 functional categories. The 5 highly down regulated synaptic plasticity genes were GRIA1 (41 & 28 fold), PPP1CA (32 and 18 fold), NGFR (23 and 16 fold), NPTX2 (18 and 27 fold) and AKT (15 and 6 fold) in clade B and C infected astrocytes, respectively. Interestingly, in this study, we have observed 27 fold down-regulation of NPTX2 gene expression in clade C infected astrocytes. Whereas in clade B infected cells, NPTX2 was down-regulated by 19 fold. Neuronal pentraxin 2 protein is also being referred to as neuronal activity-regulated pentraxin, a secreted protein with features of a calcium dependent lectin, which acts as a neurotrophic factor promoting neuronal migration, synapse formation and dendritic outgrowth of neurons with the same potency as neurotrophins and growth factors [30], [31], [32]. Down-regulation of NPTX2 may leads to decreased dendritic outgrowth and synapse formation, and thereby it may have negative regulatory effect on learning and memory. It is possible that some unexplained functions may be associated with NPTX2 gene and further studies on this gene with respect to clade B/C infections and associated neuronal activation need to be elucidated in future studies. If we see the number of genes down-regulated in clade B infected astrocytes according to their functional category, 12 immediate early response genes, 11 long term potentiation, 8 Postsynaptic density, 7 long term depression, 6 neuronal receptor, 5 CREB factors and 3 cell adhesion functional genes were down-regulated. Although each of these down-regulated genes has its own role in the development of memory, cognitive and motor functions, in this study, we have further analyzed the expression profile of three dendritic spine density regulatory proteins and further, the dendrite spine morphology changes in clade B and C infected SK-N-MC neuroblastoma cells. In this study, in PCR array, total 8 postsynaptic density regulatory genes (ADAM10, ARC, DLG4 (PSD95), GRIA1, NMDAR1, GRIN2B, GRM1, PICK1) were significantly down-regulated in clade B infected astrocytes than control and clade C infected cells. Out of these, important role of ARC, NMDAR1 and GRM1 in the maintenance of synaptic density and dendritic architecture have been well documented. An activity-regulated cytoskeleton-associated protein is an ideal candidate for regulating spine morphology and network stability [33]. Its expression is tightly regulated by neuronal activity [34], [35], and its RNA and protein are localized to dendrites and spines after activity [36], [37], [38], [39]. Furthermore, ARC induction is required for late LTP and memory consolidation [40], [41], [42]. NMDA receptor 1 is an essential gene for the maintenance of spine density [43], [44] and NR1 knockdown in cultures results in unstable spines and reduced spine density [44]. Different studies using in vitro and in vivo models support the important regulatory role of GRM1 receptors in neuronal dendritic and spine morphology [45], [46], [47]. Therefore, these three genes (ARC, NMDAR1, GRM1) were selected for further flow cytometry analysis. In the flow-cytometry analysis, we have observed that the levels of these three proteins were significantly down regulated in clade B infected cells than control and clade C infected primary human astrocytes and SK-N-MC neuroblastoma cells indicating more dendrite morphological changes in the clade B infected neuronal cells.

Further, in the confocal microscopic analysis of HIV infected SK-N-MC neuroblastoma cells, numerous changes in dendritic architecture have been observed which includes decreased synaptic density, dendrite area and diameter; loss of dendritic spines, decreased spine area and length in clade B infected SK-N-MC cells than control and clade C infected cells. Dendritic spines are tiny protrusions along dendrites, which constitute major postsynaptic sites for excitatory synaptic transmission. These spines are highly motile and can undergo remodeling even in the adult nervous system. Spine remodeling and the formation of new synapses are activity dependent processes that provide a basis for memory formation. A loss or alteration of these structures has been described in patients with neurodegenerative disorders such as Alzheimer's disease [48], [49]. Dendrite diameter also plays an important role in maximizing the effectiveness of the synaptic input [50]. Therefore, altered dendrite and spine morphology and number of spines may negatively affect the synaptic plasticity in clade B and C infected patients. In the neurodegenerative process of HIV encephalitis, synaptic and dendritic damage in the early stages that progresses to neuronal loss in the neocortex, limbic and striato-nigral systems [51], [52], [53], [54], [55], [56] has been reported. Therefore, this study indicates that HIV-1 clade B infection results in decreased number of spines and altered dendrite morphology than clade C infection that may lead to the decreased synaptic plasticity.

Apoptosis of neuronal cells in HIV-1 infected individuals relates to cognitive and motor dysfunctions [57], [58]. The factors initiating HAD seem to be pleotropic and include HIV-1 infection of certain CNS-based cell types that may compromise their natural functions and further lead to the release of viral particles or proteins that trigger apoptosis of neurons [59], [60], [61]. Alternatively, the infection of macrophages or microglia may enhance the release of pro-inflammatory cytokines such as tumor necrosis factor (TNF-α) and IFN-δ, which are toxic to neurons [62], [63]. As we have expected, induction of astrocyte cell death was significantly higher in HIV-1 clade B infected astrocytes when compared to the clade C infected cells. In conclusion, our results indicate that HIV-1 clade C is less neuropathogenic than clade B. A recent study to investigate HIV-1 clades specific neuropathogenesis using the severe combined immune deficiency (SCID) mouse HIV encephalitis model indicated that infection with HIV-1 clade C resulted in a milder cognitive dysfunction compared to HIV-1 clade B [64]. Results from clinical studies on development of HIV-associated neurocognitive impairment have suggested that HIV subtypes might differ in biological properties with respect to their capacity to cause HAND [65]. Further, it is possible that synaptic plasticity gene down-regulation, changes in dendritic architecture and induction of astrocyte apoptosis in clade B infected CNS cells compared to clade C may be responsible for increased incidence of HAND in clade B infected patients.
